# The Association Between Cardiovascular Risk Factors and Emotional States: A Summary of the Tehran Lipid and Glucose Study

**DOI:** 10.5812/ijem-167233

**Published:** 2026-05-05

**Authors:** Mahdieh Niknam, Leila Cheraghi, Proushat Shirvani, Maryam Mataji-Amirroud, Parnian Parvin, Sara Jalali-Farahani, Fereidoun Azizi, Parisa Amiri

**Affiliations:** 1 Research Center for Social Determinants of Health, Research Institute for Metabolic and Obesity Disorders, Research Institute for Endocrine Sciences, Shahid Beheshti University of Medical Sciences, Tehran, Iran; 2 Department of Epidemiology and Biostatistics, Research Institute for Endocrine Sciences, Shahid Beheshti University of Medical Sciences, Tehran, Iran; 3 Institute of Public Health and Wellbeing, University of Essex, Colchester, UK; 4 Endocrine Research Center, Research Institute for Endocrine Disorders, Research Institute for Endocrine Sciences, Shahid Beheshti University of Medical Sciences, Tehran, Iran

**Keywords:** Cardiovascular Risk Factors, Emotional States, Depression, Anxiety, Stress, Tehran Lipid and Glucose Study

## Abstract

**Context:**

The current study aimed to review the studies that investigated the association of cardiovascular risks and emotional states in men and women participating in the Tehran Lipid and Glucose Study (TLGS).

**Evidence Acquisition:**

A comprehensive search of PubMed, Scopus, and Web of Science was conducted to identify cross-sectional and prospective studies published through February 2026, based on predetermined inclusion and exclusion criteria. Cardiovascular risk factors were defined as weight status, blood pressure, and insulin resistance, while emotional states encompassed depression, anxiety, and stress. Given the heterogeneity in study designs and outcomes, the findings were synthesized narratively, and a meta-analysis was not performed.

**Results:**

Six eligible studies (four cross-sectional and two prospective) were included. Their findings indicated that hypertension and insulin resistance were associated with increased emotional distress, particularly among women with diagnosed hypertension. Long-term weight analyses showed that higher stress levels in males were associated with progression to overweight and obesity from childhood. Severe obesity was associated with greater mental distress in both sexes. Studies on obesity phenotypes indicated that higher anxiety and stress scores were observed among metabolically unhealthy obese women, a pattern also observed in metabolically unhealthy men regardless of weight status.

**Conclusions:**

Evidence from the TLGS indicated a sex-dependent association between cardiovascular risks and emotional states, with a significant association observed specifically in women.

## 1. Context

Cardiovascular diseases (CVDs) are responsible for over 20.5 million fatalities annually (1, 2). This issue is particularly severe in developing countries, including Iran, where urbanization and lifestyle changes have elevated cardiovascular risk factors (3, 4, 5, 6). Despite advancements in clinical management, the growing impact of CVDs highlights the need for a thorough investigation into determinants beyond traditional risk factors (7).

Epidemiological research highlights the significant association between psychological factors, CVD, and related risk factors (8, 9, 10, 11, 12). In light of this evidence, the American Heart Association (AHA) recommends recognizing psychiatric disorders as major risk factors for coronary heart disease, on par with conditions like hyperlipidemia, diabetes, hypertension, and smoking (13, 14). In this regard, research indicates a strong association between depression and cardiovascular risks, with individuals suffering from depression compared to the general population being twice as likely to manifest myocardial infarction (15, 16). Additionally, patients with coronary artery disease who also have depression face a threefold higher risk of developing dementia (17). These investigations demonstrate a complex relationship in which negative emotional states increase cardiovascular risk through physiological mechanisms, including chronic stress responses, elevated sympathetic nervous system activity, and inflammatory processes (18). This interplay ultimately exacerbates issues such as glucose-, blood pressure (BP)-dysregulation, atherosclerosis progression, and cardiac arrhythmias (19).

Given the significant impact of emotional health on the behavioral and biological mediators of CVD (20), a comprehensive understanding of these associations is crucial for developing tailored rehabilitation programs aimed at mitigating adverse outcomes across diverse populations.

The Tehran Lipid and Glucose Study (TLGS), a large-scale cohort study, encompassing over 15,000 individuals aged ≥ 3 years in a representative Tehran population, has uniquely elucidated the epidemiology of cardiovascular risk factors in Iran, a region undergoing intense nutritional and epidemiological transitions (21). TLGS’s extensive longitudinal dataset includes comprehensive assessments of metabolic parameters, anthropometric trajectories, socioeconomic status, and crucially, emotional and psychological well-being, thereby providing an exceptional platform to unravel the intricate interrelations between cardiovascular risk and mental health outcomes (22). The current narrative review provides a focused summary of findings from the TLGS related to the association between cardiovascular risk factors and emotional states.

## 2. Evidence Acquisition

### 2.1. Data Source

The TLGS study has two main phases: the first from Feb. 1999 to Aug. 2001, and the second involving six assessments every three years from 2002 to 2021 (21, 22). Initially, psychosocial variables were introduced during the fifth follow-up examination (2014, 2015, 2016). Considering that participants had previously responded to detailed inquiries regarding their behavioral and physical health traits, the TLGS committee decided to evaluate psychological aspects using validated questionnaires distributed to smaller, randomly chosen groups. Hence, participants were randomly assigned to three groups for psychological assessments, and one group completed an emotional state information. However, in the last follow-up assessment (2018, 2019, 2020, 2021), data on emotional states were comprehensively gathered from all adult participants. To evaluate emotional states, DASS-21 was employed (23). This self-reported questionnaire measures the extent of depression, anxiety, and stress of respondents over the past week. It consists of three subscales, each containing seven items. Respondents evaluate their experiences using a four-point scale. Subscale scores are determined by summing the relevant item scores and multiplying the total by two. The standard ranges are 0 - 9 for depression, 0 - 7 for anxiety, and 0 - 14 for stress.

### 2.2. Search Strategy and Study Selection

All English-language studies, until February 2026, investigating cross-sectional or prospective studies with these keywords in the title, abstract, and text: cardiovascular risks, weight status, BP status, insulin resistance, triglyceride-glucose (TyG) index, metabolic risk, emotional states, depression, anxiety, and stress in the framework of the TLGS (in adults), across multiple databases including PubMed, Scopus, and Web of Science. Two reviewers critically evaluated all papers independently and data was extracted by one reviewer and rechecked by a second one. Furthermore, given the heterogeneity of study designs and outcomes, a formal meta-analysis was not feasible; therefore, findings are summarized narratively.

### 2.3. Cardiovascular Risks: Definition and Assessment

The study included several cardiovascular risk factors: weight status, assessed through body mass index (BMI kg/m²) and waist circumference (WC); BP status; and insulin resistance, measured using the triglyceride-glucose (TyG) index (24). BP readings were collected following standardized protocols. Trained personnel measured BP twice, at five-minute intervals using a calibrated mercury sphygmomanometer. The average of the two readings was used to determine systolic blood pressure (SBP) and diastolic blood pressure (DBP). Information regarding the history of hypertension and medication was also provided. Hypertension was defined by the following criteria (25): 1) An average SBP of at least 130 mmHg, or 2) An average DBP of at least 80 mmHg, or 3) Self-reported use of antihypertensive medication.

The TyG index is a simple, cost-effective, and valid proxy of insulin resistance. It is calculated using the formula: TyG index = ln (FTG (mg/dL) × FBS (mg/dL))/2 (26). Fasting blood sugar (FBS) and fasting triglycerides (FTG) were assessed using a specific laboratory method, following a fasting period of 12 to 14 hours (27, 28).

## 3. Results

Following a comprehensive review of the literature, six studies (4 cross-sectional, 2 prospective) were included (Table 1). All statistical estimates (e.g., regression coefficients, odds ratios, and p values) are reported as presented in the original studies, and no quantitative synthesis or re-analysis was performed in this narrative review.

**Table 1.** Summary of Included Studies

Study (Year)	TLGS Phase (Years)/Study Type	Sample Size	Age Range/Mean Age	Exposure (s)	Outcome (s)	Main Adjustments	Key Findings
**Mehrabi and Amiri, 2022 (29)**	Phase VI (2014, 2015, 2016)/ Cross-sectional	2,272 adults	20 - 87 y; mean ≈ 49 y	Socio-demographic, behavioral, and clinical factors (age, education, marital status, smoking, obesity, chronic disease)	Depression, anxiety, stress (DASS-21)	Multivariable sex-specific models including age, education, employment, marital status, smoking, physical activity, obesity, chronic disease	Women reported higher depression, anxiety, and stress scores. Smoking was associated with all emotional states in both sexes. Obesity was associated with depression, anxiety, and stress in men, but only stress in women. Older age and higher education were generally protective.
**Mehrabi and Amiri., 2021 (30)**	Phase VI (2016, 2017, 2018, 2019)/ Cross-sectional	2,469 (1,158 men; 1,311 women)	≥ 20 y; mean ≈ 46 y	Obesity phenotypes (MHNO, MHO, MUNO, MUO based on BMI and metabolic status)	Depression, anxiety, stress (DASS-21)	Age, marital status, education, job status, smoking, physical activity	Metabolically unhealthy phenotypes were associated with higher anxiety and stress in both sexes. MUO women and metabolically unhealthy men showed significantly higher anxiety and stress. No obesity phenotype was associated with depression after adjustment.
**Mahani et al., 2022 (31)**	Phases I - VI (1999, 2000, 2001, 2002, 2003, 2004, 2005, 2006, 2007, 2008, 2009, 2010, 2011, 2012, 2013, 2014, 2015, 2016, 2017, 2018, 2019)/ Prospective	687 offspring	Childhood 4 - 18 y → young adulthood 22 - 36 y	BMI trajectories from childhood (healthy weight vs. persistent increasing overweight/obesity)	Depression, anxiety, stress in young adulthood (DASS-21)	Age, education, smoking status, chronic diseases (young adulthood)	Persistent increasing overweight/obesity trajectory was associated with higher stress levels in young adult males. No significant associations were observed with depression or anxiety in either sex or stress in females.
**Amiri et al., (32)**	Phases II - VII (2002, 2003, 2004, 2005, 2006, 2007, 2008, 2009, 2010, 2011, 2012, 2013, 2014, 2015, 2016, 2017, 2018, 2019, 2020, 2021)/ Prospective	5,550 adults (2,308 men; 3,242 women)	≥ 20 years at baseline; mean age: men 42.4 ± 13.6, women 40.6 ± 12.7 y	Long-term multi-trajectories of BMI and waist circumference (WC), classified into four ascending trajectory groups	Depression, anxiety, and stress (DASS-21) at last follow-up	Age, marital status, education level, employment status, smoking, physical activity, and chronic diseases	Severe obesity with central obesity trajectories were associated with higher anxiety and/or depression, particularly among university-educated individuals, unemployed participants, unmarried men, and married women
**Niknam et al., 2024 (11)**	Phase VI (2014, 2015, 2016, 2017)/ Cross-sectional	HRQoL: 7,257; Emotional states: 2,499	≥ 20 y; mean ≈ 47 y	Blood pressure status (normotensive, undiagnosed HTN, diagnosed HTN with vs. without treatment adherence)	HRQoL (SF-12 PCS & MCS), depression, anxiety, stress (DASS-21)	Age, BMI, chronic disease, occupation, education, marital status, smoking, physical activity	Treatment adherence was inversely associated with physical HRQoL in both sexes. Poor adherence was linked to worse mental HRQoL in women. Higher treatment adherence was associated with anxiety in women, whereas poor adherence was associated with depression and stress. Undiagnosed hypertension showed no significant HRQoL or emotional deficits.
**Shirvani et al., 2026 (33)**	Phase VII (2018, 2019, 2020, 2021)/ Cross-sectional	5,379 (2,505 men; 2,874 women)	≥ 20 y; mean ≈ 43 y	Triglyceride–glucose (TyG) index; BP status (normotensive, suspected HTN, diagnosed HTN)	Depression, anxiety, stress (DASS-21)	Age, occupation, education, marital status, smoking, physical activity, BMI, CHD, CKD	Higher TyG index was positively associated with depression, anxiety, and stress only in women with diagnosed hypertension. No significant associations were observed in men or other BP groups.

### 3.1. Factors Associated with Depression, Anxiety, and Stress in Men and Women

**Figure 1.**
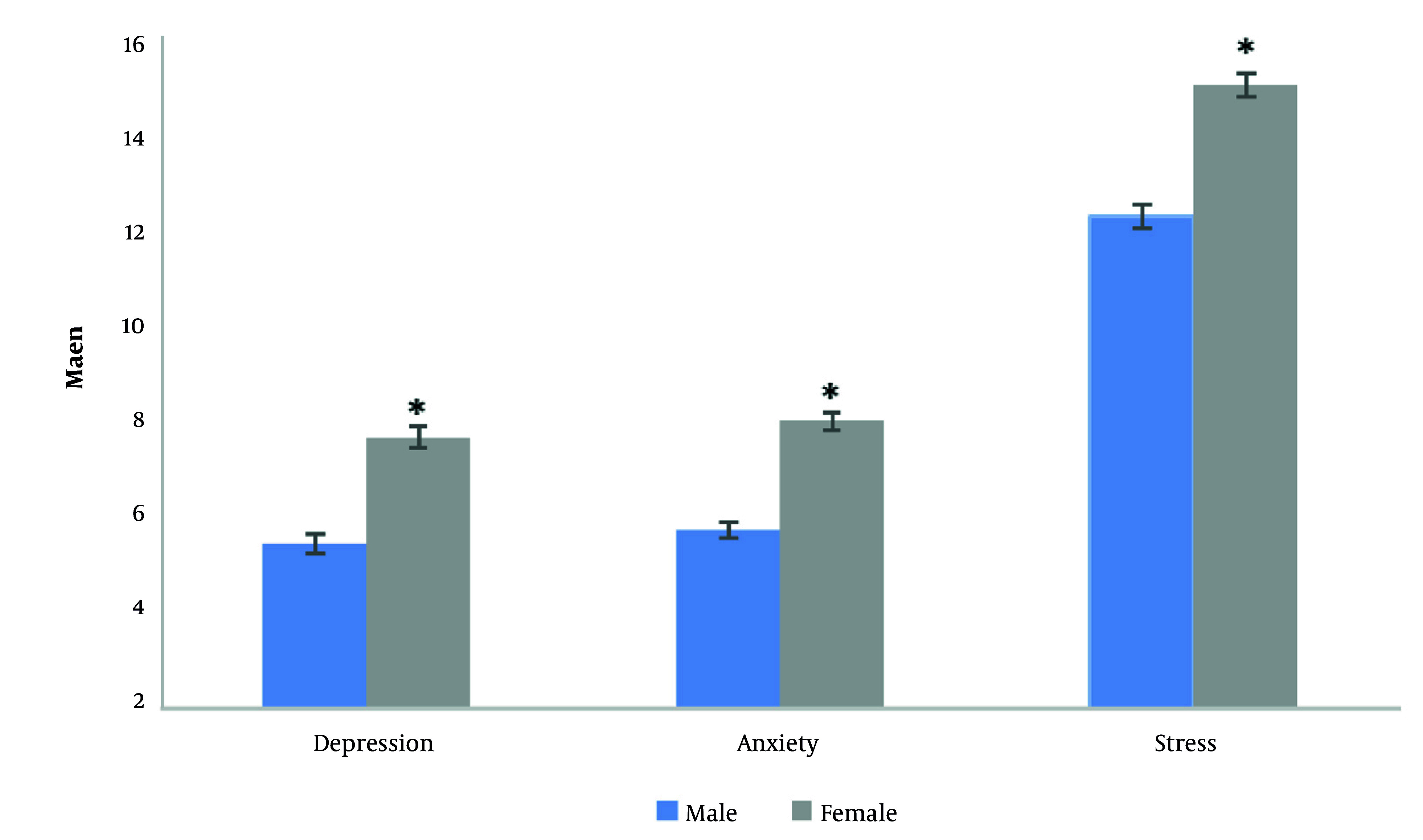
The depression, anxiety, and stress means ± SE among men and women (* P < 0.01).

In the original cross-sectional study involving 2,272 participants (55.72% women) with a mean age of 47.23 years in the fifth follow-up examination of the TLGS (2014, 2015, 2016) (29) revealed that women indicated the considerable higher levels of DASS compared to men (Figure 1). Lower levels of psychological distress were observed with increasing age among both males and females, while higher educational attainment was associated with depression. Lower levels of depression were observed among married men, whereas this association was less evident among women. Higher DASS scores were reported among unemployed men compared with employed men, with no corresponding association observed among women. Obesity (BMI ≥30.0 kg/m²) was associated with stress in women as well as depression and anxiety in men. Interestingly, no significant association was found between physical activity and psychological health outcomes; Smoking was associated with higher psychological distress scores in men and women, particularly among hookah smoker female, who displayed notably higher anxiety and stress (Table 2).

**Table 2.** The Adjusted Association Between the Participants' Characteristics and the Emotional States According to Their Gender ^a, b^

Variables	Men (n = 1006)						Women (n = 1266)					
	Depression		Anxiety		Stress		Depression		Anxiety		Stress	
	β (SE)	P-Value	β (SE)	P-Value	β (SE)	P-Value	β (SE)	P-Value	β (SE)	P-Value	β (SE)	P-Value
**Age (y)**	-0.07 (0.01)	< 0.001	-0.08 (0.01)	< 0.001	-0.14 (0.01)	< 0.001	-0.06 (0.20)	0. 01	-0.07 (0.02)	0.001	-0.12 (0.02)	< 0.001
**Education level**												
Illiterate/primary	(Ref)	(Ref)	(Ref)	(Ref)	(Ref)	(Ref)	(Ref)	(Ref)	(Ref)	(Ref)	(Ref)	(Ref)
Secondary	-1.55 (0.51)	0.003	-1.01 (0.47)	0.03	-0.05 (0.64)	0.10	-1.55 (0.70)	0.02	-2.29 (0.08)	0.004	-1.09 (0.83)	0.19
Higher	-2.79 (0.34)	< 0.001	-1.85 (0.53)	0.01	-2.41 (0.73)	0.001	-3.58 (0.89)	< 0.001	-1.01 (0.63)	0.11	-3.06 (0.73)	0.004
**Employment **												
Yes	(Ref)	(Ref)	(Ref)	(Ref)	(Ref)	(Ref)	(Ref)	(Ref)	(Ref)	(Ref)	(Ref)	(Ref)
No	1.67 (0.34)	< 0.001	1.53 (0.31)	< 0.001	1.64 (0.43)	< 0.001	0.17 (0.67)	0.79	0.11 (0.60)	0.84	0.10 (1.27)	0.93
**Marital status**												
Single	(Ref)	(Ref)	(Ref)	(Ref)	(Ref)	(Ref)	(Ref)	(Ref)	(Ref)	(Ref)	(Ref)	(Ref)
Married	-0.99 (0.05)	0.05	0.59 (0.46)	0.20	0.22 (0.64)	0.72	-0.75 (0.77)	0.33	0.69 (0.69)	0.32	0.53 (0.92)	0.56
Divorced/Widowed	1.1 (0.81)	0.17	2.16 (0.74)	0.003	0.65 (1.02)	0.52	0.69 (1.07)	0.51	1.69 (0.96)	0.08	0.10 (1.27)	0.93
**Physical activity**												
Low	(Ref)	(Ref)	(Ref)	(Ref)	(Ref)	(Ref)	(Ref)	(Ref)	(Ref)	(Ref)	(Ref)	(Ref)
Moderate	-0.04 (0.04)	0.31	0.21 (0.36)	0.56	-0.34 (0.50)	0.49	-0.92 (0.56)	0.10	0.05 (0.52)	0.91	-0.95 (0.68)	0.16
High	-0.65 (0.36)	0.07	0.05 (0.44)	0.86	-0.05 (0.46)	0.27	-0.92 (0.57)	0.11	0.08 (0.50)	0.87	-1.02 (0.66)	0.12
**Cigarette Smoking **												
No	(Ref)	(Ref)	(Ref)	(Ref)	(Ref)	(Ref)	(Ref)	(Ref)	(Ref)	(Ref)	(Ref)	(Ref)
Yes	0.94 (0.48)	0.05	0.89 (0.44)	0.04	1.40 (0.60)	0.20	2.31 (1.22)	0.05	2.37 (1.10)	0.03	3.77 (1.45)	0.01
**Hookah smoking**												
No	(Ref)	(Ref)	(Ref)	(Ref)	(Ref)	(Ref)	(Ref)	(Ref)	(Ref)	(Ref)	(Ref)	(Ref)
Yes	-0.01 (0.49)	0.97	0.56 (0.44)	0.21	0.78 (0.61)	0.20	0.99 (0.92)	0.27	1.89 (0.83)	0.02	2.74 (1.09)	0.02
**Chronic diseases**												
No	(Ref)	(Ref)	(Ref)	(Ref)	(Ref)	(Ref)	(Ref)	(Ref)	(Ref)	(Ref)	(Ref)	(Ref)
Yes	0.15 (0.37)	0.67	0.93 (0.34)	0.006	0.78 (0.47)	0.09	0.08 (0.55)	0.88	0.98 (0.49)	0.04	0.34 (0.65)	0.60
**Obese**												
No	(Ref)	(Ref)	(Ref)	(Ref)	(Ref)	(Ref)	(Ref)	(Ref)	(Ref)	(Ref)	(Ref)	(Ref)
Yes	0.72 (0.34)	0.03	0.81 (0.31)	0.009	1.30 (0.43)	0.003	0.75 (0.49)	0.12	0.79 (0.44)	0.07	1.67 (0.59)	0.005

^a^ The estimates were derived using multiple linear regression analysis with emotional states as dependent variables. In this model having an illiterate or primary education, being employed, being single, having low physical activity, not having cigarette or hookah smoking, not having any chronic disease and not being fat were considered as the reference category.

^b^ Physical activity levels were determined as low, moderate, and high (< 600, 600 - 3000, > 3000 METmin/week, respectively).

The findings on the association between cardiovascular risk factors and emotional states are as follows:

### 3.2. Weight Status and Emotional States

A cross-sectional study by Mehrabi Mehrabi and Amiri, utilizing data from the fifth follow-up examination of the TLGS, defined various obesity phenotypes and investigated their association with emotional states, specifically depression, anxiety, and stress, among 2,469 adults (1,158 men and 1,311 women) (30). Four obesity phenotypes were identified among participants: non-obese without metabolic syndrome, accounting for 45.9%; non-obese with metabolic syndrome, comprising 23.6%; Obese without metabolic syndrome, representing 20.9%; and Obese with metabolic syndrome, making up 9.6%. The study reported that the likelihood of experiencing elevated anxiety levels was significantly associated with Obese with metabolic syndrome (Odds Ratio [OR]: 1.78; 95% CI: 1.25 - 2.54) and non-obese with metabolic syndrome men (OR: 1.61; 95% CI: 1.17 - 2.22) when compared to non-obese men without metabolic syndrome. Similarly, Obese women with metabolic syndrome showed a greater likelihood of heightened anxiety (OR: 1.73; 95% CI: 1.28 - 2.34) relative to non-obese women without metabolic syndrome phenotype. Furthermore, the odds of elevated stress were considerably associated with non-obese with metabolic syndrome phenotype in men (OR: 1.40; 95% CI: 1.02 - 1.90) as well as Obese women with metabolic syndrome (OR: 1.45; 95% CI: 1.07 - 1.96) compared to their non-obese counterparts without metabolic syndrome. Additionally, a noticeable difference in higher depression levels was found in Obese women with metabolic syndrome prior to adjustments (OR: 1.39; 95% CI: 1.04 - 1.84), although no significant differences were illustrated in either sex after adjustments (Table 3).

**Table 3.** The Odds Ratios and 95% Confidence Intervals for Depression, Anxiety and Stress Among Men and Women (n = 2469) ^a^

Gender and Status	Depression		Anxiety		Stress	
	OR (95%CI)	P-Value	OR (95%CI)	P-Value	OR (95%CI)	P-Value
**Male**						
Non-obese without MetS	(Ref)		(Ref)		(Ref)	
Non-obese with MetS	1.21(0.79 - 1.84)	0.31	1.61(1.17 - 2.22)	0.003	1.40(1.02 - 1.90)	0.04
Obese without MetS	1.40(0.80 - 2.44)	0.24	1.22(0.74 - 2.04)	0.43	1.26(0.78 - 2.02)	0.34
Obese with MetS	1.20(0.79 - 1.84)	0.39	1.78(1.25 - 2.54)	<0.001	1.34(0.95 - 1.90)	0.09
**Female**						
Non-obese without MetS	(Ref)		(Ref)		(Ref)	
Non-obese with MetS	0.80(0.56 - 1.16)	0.24	1.16(0.84 - 1.61)	0.36	1.04(0.75 - 1.46)	0.80
Obese without MetS	0.94(0.64 - 1.40)	0.77	1.13(0.80 - 1.61)	0.50	1.26(0.89 - 1.78)	0.20
Obese with MetS	1.10(0.78 - 1.53)	0.60	1.73(1.28 - 2.34)	<0.001	1.45(1.07 - 1.96)	0.02

^a^ Adjusted for age, marital status (Ref = married), level of education (Ref = higher), job status (Ref = employed) and level of physical activity (Ref = high).

**Figure 2.**
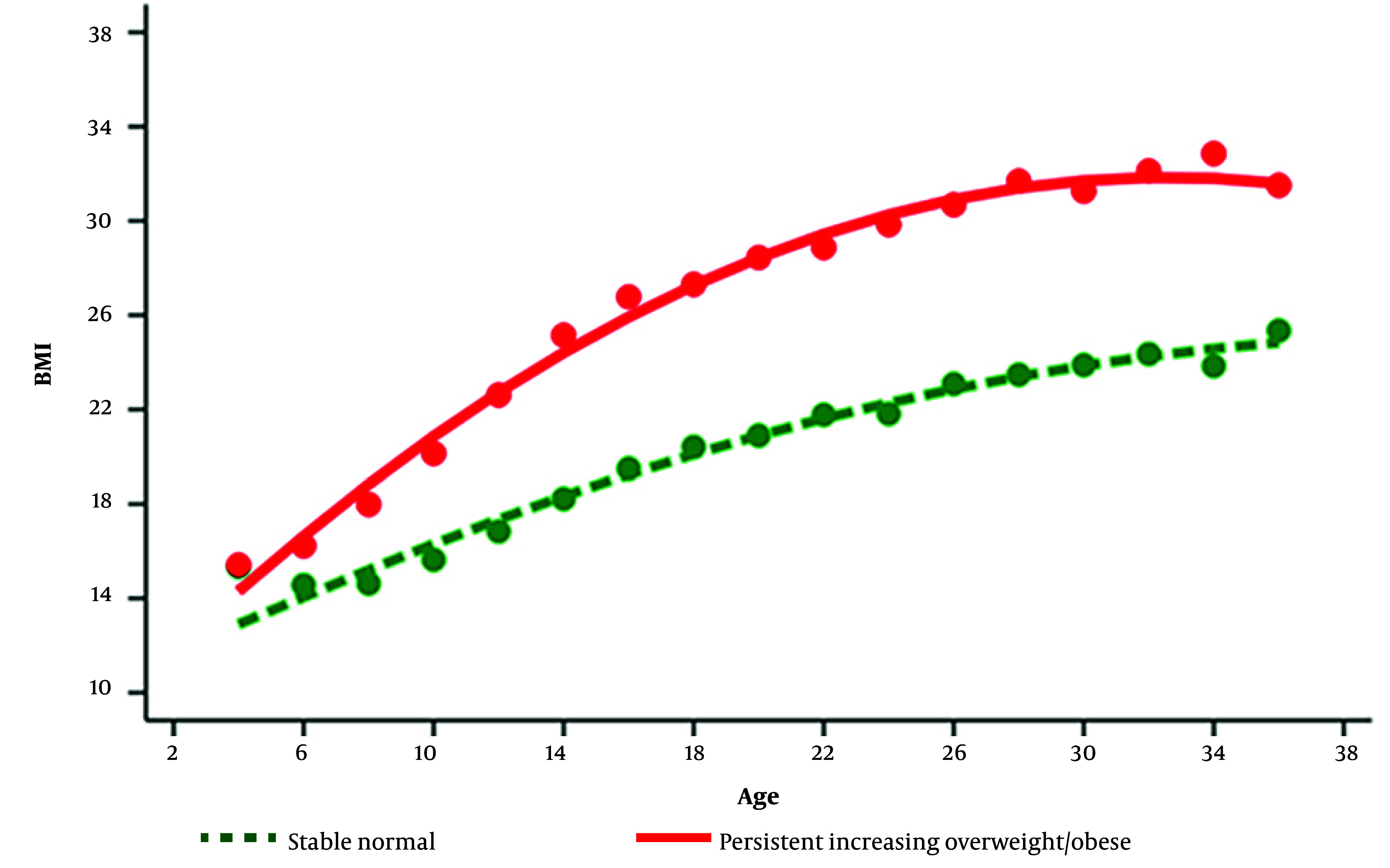
The distinct Body Mass Index (BMI) trajectories from the first phase (children aged 4 to 18 years) to the sixth phase (young adults aged 22 to 36 years) are illustrated. The lines represent class-specific mean predicted BMI levels as a function of age, estimated using the best-fitting group-based model—stable normal weight (SNW: dashed line) and persistent increasing overweight/obesity (PIO: continuous line). Overweight and obesity were determined based on BMI thresholds established by the World Health Organization. For children aged 4 to 18 years, these thresholds were averaged across sexes for each age. In adults aged 19 years and older, a BMI of ≥ 25 indicated overweight, while a BMI of ≥ 30 signified obesity.

Based on the findings derived from obesity phenotypes, two studies further examined the association between long-term weight status and emotional states within the TLGS. In this context, Mahani et al. (2022) conducted a prospective study examining the association between childhood BMI trajectories and emotional outcomes in young adulthood (31). This longitudinal study tracked 687 children (4 - 18 years), over an 18-year period, measuring their BMI every three years and evaluating mental health status in young adulthood (22 - 36 years). Overweight and obesity among children aged 4 - 18 were determined using age- and sex-specific BMI thresholds established by the WHO, with BMI percentiles (34), and BMI levels for individuals ≥ 19 years. Finally, two trajectories were determined: a Healthy Weight (HW) group (69.6%) and a Persistent Increasing Overweight/Obesity (PIO) group (30.4%) (Figure 2). The study reported that the PIO group men had a considerably higher likelihood of indicating stress compared to those in the HW group. The results showed no association between the identified trajectories and depression or anxiety in either gender.

**Figure 3.**
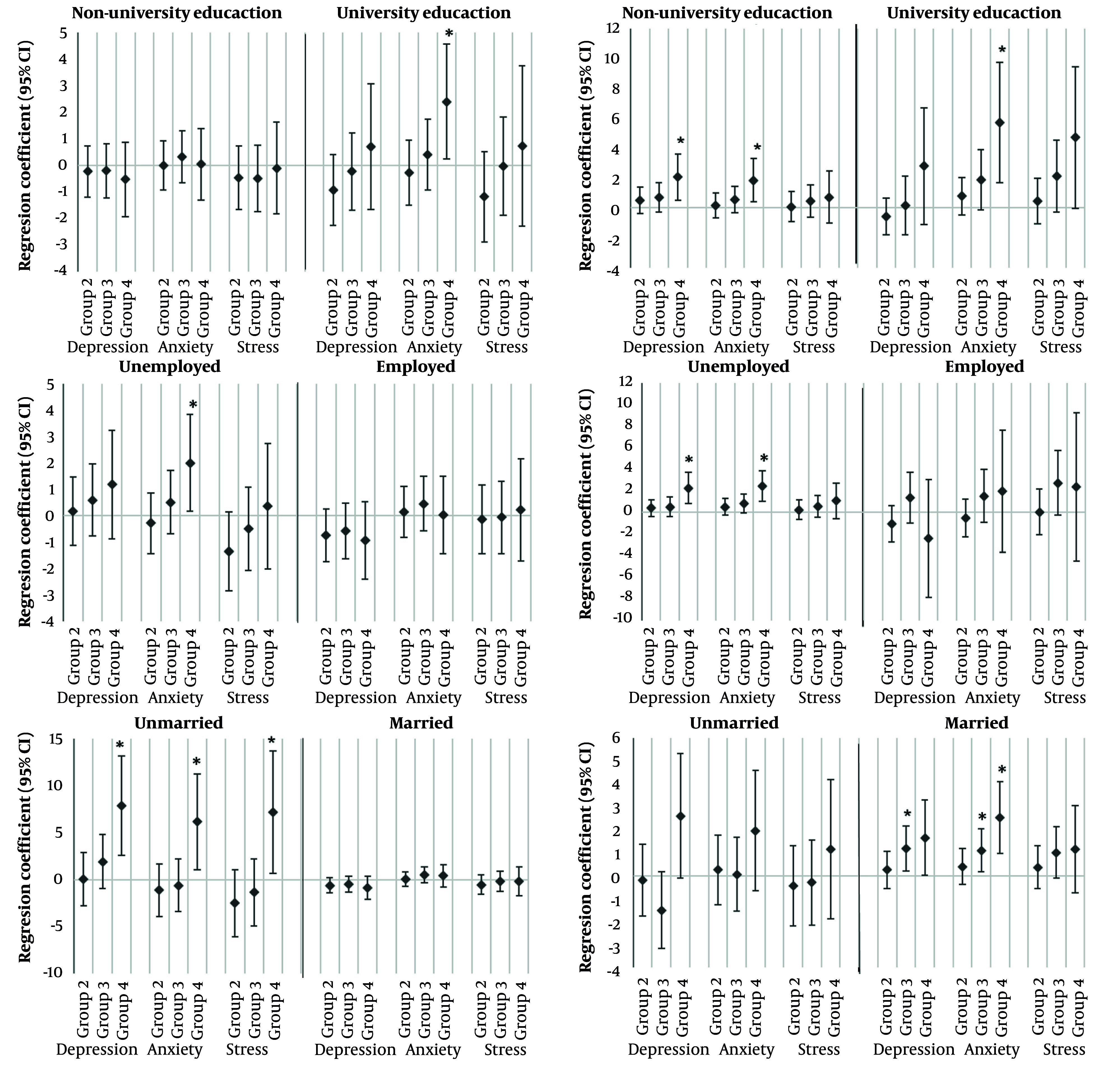
The association between BMI and waist multi-trajectory and DASS score according to sex and sociodemographic characteristics. * P < 0.05. CI, confidence interval. For each depression, anxiety, and stress variable, the regression coefficient represents the difference in mean score for each BMI and waist trajectory group compared with group 1.

Due to the limited data on long-term changes in weight status among adults and their psychological outcomes, as well as the significant influence of socio-demographics on individuals' perceived emotional states (29), recent cross-sectional study investigated the association between multiple trajectories of BMI and WC from 2002 to 2021 (32). The study involved 5,550 adults (2,308 males) recruited during the 7th phase of the TLGS, taking into account socio-demographics. The study identified four distinct trajectory groups for both men and women, labeled Group 1 to Group 4. As the trajectory group progressed from Group 1 to Group 4, both BMI and WC trends showed a worsening pattern. The study reported that university-educated males (p= 0.031) and females (p= 0.005) in Group 4 exhibited more anxiety scores compared to Group 1. Non-university-educated women in Group 4 also showed increased depression (p= 0.009) and anxiety scores (p= 0.012). Among unemployed men, Group 4 had a higher anxiety score, while unemployed women in Group 4 reported elevated depression and anxiety. Employed participants did not show significant associations with BMI and WC trajectories and emotional states. Unmarried men had significantly higher emotional state scores in Group 4 compared to Group 1, while married women in Groups 3 and 4 exhibited higher mean depression (β=1.20 and β=1.65) and anxiety scores (β=1.11 and β=2.55) compared to Group 1 (Figure 3).

### 3.3. Blood Pressure and Emotional States

The association between BP and common psychological conditions was explored by Niknam and Zolfagharypoor, utilizing the sixth phase of TLGS (11). This cross-sectional study assessed this association by considering participants’ awareness of having hypertension and their treatment adherence. Individuals aged 20 and above, who had complete data regarding their emotional states and a range of covariates (including anthropometric measurements, socio-demographic information, behavioral aspects, and clinical background), were considered, leading to a final analysis involving 2,499 individuals. Hypertension was defined as follows (35): 1) normotensive—individuals with normal BP and no history of high BP, and no taking hypertension lowering drugs; 2) undiagnosed hypertension— subjects with high BP (≥ 140/90 mmHg), without hypertension history; 3) diagnosed, committed to treatment— patients with known hypertension and taking related drugs regularly; and 4) diagnosed, non-committed to treatment— hypertensive patients with low adherence to drugs.

The study reported that women scored higher than men on the DASS-21 subscales, with mean depression scores in diagnosed hypertensive females and elevated anxiety and stress levels across all defined groups. In women, depression and anxiety significantly differed among BP groups, with normotensive females reporting the lowest, and those with poor medication adherence showing the highest scores. The association between mental health and BP statuses did not show significant differences among males. However, in women, good adherence to antihypertensive medications led to a 1.44 increase in anxiety scores. Poor adherence was associated with higher depression and stress scores, with β values of 4.65 and 5.67, respectively.

### 3.4. Insulin Resistance and Emotional States

A recent cross-sectional TLGS study evaluated the relationship between the TyG index and emotional states, taking into account individuals' BP status (33). This study analyzed data from 5,379 adult participants (53.43% female) recruited during the seventh phase of the TLGS from 2018 to 2021. Participants were categorized into three groups: the normotensive group— individuals with normal SBP and DBP and no history of hypertension; the suspected hypertension group—individuals with BP exceeding 130/80 mmHg who had not been diagnosed by a healthcare professional; and the diagnosed hypertension group—those diagnosed with hypertension or currently on antihypertensive medication, regardless of their BP readings at the time of assessment. Results showed an increasing trend in anxiety scores was noted in both genders from the normotensive to the diagnosed hypertension groups. The study notably uncovered a significant and direct positive association between emotional states and the TyG index in females diagnosed with hypertension, indicating that a one-point increase in the TyG index corresponded to an approximate three-point increase in mental distress score.

## 4. Discussion

These findings from their studies suggest that emotional distress is generally higher in women compared to men. Hypertension and insulin resistance are associated with increased emotional distress, specifically in women diagnosed with hypertension. Analysis of long-term weight status reveals that the development of overweight and obesity from childhood is associated with elevated stress levels in males. In contrast, multiple trajectories of BMI and WC highlight a relationship between severe obesity and heightened mental distress in both genders. Furthermore, our investigation into obesity phenotypes demonstrates that higher levels of anxiety and stress are associated with metabolically unhealthy obese women, while this association is similarly observed in metabolically unhealthy men, regardless of their weight status.

Globally, the higher levels of DASS observed in women compared to men are among the most extensively documented phenomena in psychology (36, 37). The more emotional and cognitive vulnerability of women can be explained by a combination of biological, psychological, social, and experiential factors. Hormonal fluctuations during the life cycles—menstrual cycle, pregnancy, and menopause— significantly influence mood regulation, as well as differences in brain structure and cognitive styles—such as a tendency toward rumination—can exacerbate emotional distress in women (11, 38). Additionally, societal pressures and expectations related to gender roles, along with the challenges of balancing work and family responsibilities, may contribute to heightened stress levels in women. Furthermore, women are more likely to experience certain traumas, including sexual violence, which increases their susceptibility to anxiety, stress, and depression compared to men (39).

The results from TyG–DASS study indicated that hypertension and insulin resistance are associated with increased emotional distress exclusively in women diagnosed with hypertension. Supporting these findings, the BP–DASS study highlights the sex-specific nature of emotional distress, revealing that higher anxiety scores are associated with diagnosed hypertension who exhibit better medication adherence in women, while higher depression and stress scores are observed among those with poor adherence. Notably, undiagnosed hypertension was not associated with emotional distress in either sex, underscoring the importance of diagnosis and awareness rather than BP levels alone (11). Sex-based variations in the associations between emotional conditions, insulin resistance, and blood pressure could emerge from biological and hormonal differences. For instance, more health-conscious women may experience varying serotonin levels, which could increase their vulnerability to anxiety and depression (40). Hormonal fluctuations throughout a woman’s life can also affect mood and emotional stability (41). Cultural norms often lead men to suppress emotions, while women are encouraged to express them, strengthening the associated with emotional states and physiological responses. Additionally, women may engage more in vigilant health monitoring when aware of their hypertension, which could negatively affect their emotional well-being (42).

The results from Mahani’s study indicated that continuously increasing overweight or obesity from early childhood is associated with elevated stress levels in young adulthood, specifically in men, but not in women. Several hypotheses may account for this observation. One possibility is that societal expectations and traditional masculine norms exert greater pressure on men to conform to ideals of physical strength and fitness, resulting in increased stress when these standards are not met (43). Additionally, men may utilize specific coping mechanisms, often avoiding social support, which could exacerbate stress related to body image (44). Biological factors, including hormonal differences that affect stress responses, may also contribute to this phenomenon. Furthermore, developmental differences in how boys and girls navigate peer dynamics and life transitions could further influence this disparity (45). Lastly, the presence of comorbid mental health issues in men may intensify their stress experiences associated with obesity (46).

The results on the multiple BMI-waist trajectories study indicate that mental health issues are heightened in both adult men and women, particularly in cases of severe obesity. The significant association can be attributed to societal stigma and discrimination, body image concerns, and biological factors such as inflammation and hormonal changes that may affect mood regulation in individuals experiencing severe obesity (47, 48). Additionally, the findings regarding obesity phenotypes revealed that while levels of anxiety and stress are observed among metabolically unhealthy obese women, this association is similarly observed in metabolically unhealthy men, regardless of their weight status. This highlights the importance of metabolic health in understanding mental health challenges across different genders and suggests that targeted interventions should focus on metabolic profiles in addition to weight status.

The current narrative review summarizes findings from the TLGS regarding the association between cardiovascular risk factors and emotional states, offering a comprehensive overview of how weight status, BP, and insulin resistance are associated with common psychological conditions within a Middle Eastern population. However, the generalizability of these results is constrained by the absence of data from rural and suburban populations in Iran.

## 5. Conclusions

The studies included in the review consistently demonstrate that cardiovascular risks are closely associated with emotional states in a sex-dependent manner, with a stronger association observed in women. Further research involving diverse populations across Iran appears crucial to validate these findings.
